# Measuring continuity of care in general practice: a comparison of two methods using routinely collected data

**DOI:** 10.3399/BJGP.2022.0043

**Published:** 2022-08-23

**Authors:** Sally A Hull, Crystal Williams, Peter Schofield, Kambiz Boomla, Mark Ashworth

**Affiliations:** Wolfson Institute of Population Health, Queen Mary University of London, London.; Wolfson Institute of Population Health, Queen Mary University of London, London.; School of Population Health and Environmental Sciences, King’s College London, London.; Wolfson Institute of Population Health, Queen Mary University of London, London.; School of Population Health and Environmental Sciences, King’s College London, London.

**Keywords:** continuity of patient care, health surveys, primary health care, routinely collected health data

## Abstract

**Background:**

Despite well-documented clinical benefits of longitudinal doctor–patient continuity in primary care, continuity rates have declined. Assessment by practices or health commissioners is rarely undertaken.

**Aim:**

Using the Usual Provider of Care (UPC) score this study set out to measure continuity across 126 practices in the mobile, multi-ethnic population of East London, comparing these scores with the General Practice Patient Survey (GPPS) responses to questions on GP continuity.

**Design and setting:**

A retrospective, cross-sectional study in all 126 practices in three East London boroughs.

**Method:**

The study population included patients who consulted three or more times between January 2017 and December 2018. Anonymised demographic and consultation data from the electronic health record were linked to results from Question 10 (‘seeing the doctor you prefer’) of the 2019 GPPS.

**Results:**

The mean UPC score for all 126 practices was 0.52 (range 0.32 to 0.93). There was a strong correlation between practice UPC scores measured in the 2 years to December 2018 and responses to the 2019 GPPS Question 10, Pearson’s *r* correlation coefficient, 0.62. Smaller practices had higher scores. Multilevel analysis showed higher continuity for patients ≥65 years compared with children and younger adults (β coefficient 0.082, 95% confidence interval = 0.080 to 0.084) and for females compared with males.

**Conclusion:**

It is possible to measure continuity across all practices in a local health economy. Regular review of practice continuity rates can be used to support efforts to increase continuity within practice teams. In turn this is likely to have a positive effect on clinical outcomes and on satisfaction for both patients and doctors.

## INTRODUCTION

Longitudinal continuity of care, repeated contacts between a patient and the same doctor, is known to have important benefits for patients, for doctors, and for health systems. Recent research has demonstrated an association between higher rates of continuity and reduced all-cause mortality;^1^^,^^2^ this was found across both primary and secondary care, and in a range of health systems across Europe. Other positive outcomes for patients include greater patient satisfaction with services,^3^ improved adherence to medical advice and uptake of preventive medicine,^4^^,^^5^ lower use of hospital care,^6^ and a reduction in overall healthcare costs.^7^

Longitudinal continuity, which is one aspect of relationship-based care, is highly valued by doctors, particularly for patients with serious, complex, or psychological problems,^8^ and is frequently reported as one of the core factors that makes the work of a GP rewarding.^9^

Despite these benefits there has been a steady decline in measures of continuity for patients across general practice in England between 2012 and 2017.^10^^,^^11^ Using data from the annual General Practice Patient Survey (GPPS), which has been used in the UK since 2008 to gain user views on a range of practice services,^12^ studies find lower rates of continuity in urban and deprived populations, but a similar decline in continuity across all groups and geographical settings over the study period.^10^ Reasons given for this decline include the expansion of larger practices and the prioritisation of rapid access over continuity,^13^ GPs increasingly working part time,^10^ and difficulties recruiting GPs, which in turn has led to higher list sizes and workload.^14^

There are a variety of ways of conceptualising continuity of care. The most widely used models distinguish between longitudinal relationship continuity with a regular doctor, and management continuity that is necessary to share information and provide a seamless service between providers of care.^15^^,^^16^ Although longitudinal continuity does not necessarily translate into the patient experience of a caring relationship with a trusted doctor, it is a necessary precondition. For the purposes of this study the assessment of longitudinal continuity is used as a proxy for a well-functioning doctor–patient partnership.

The most common measure of continuity is the Usual Provider of Care (UPC) index^16^^–^^18^ which measures the proportion of contacts with the most regularly seen doctor during a specified time period. The alternative Bice–Boxerman method^19^ makes allowance for the distribution of contacts a patient has with different GPs, but is less intuitive for clinicians to understand. Both these methods require measurement over a prolonged time period (1 or 2 years) in contrast with the recently developed St Leonard’s Index of Continuity of Care,^20^ which can be used for monthly audit in practices with personal list systems. The UPC was selected for this study as it is straightforward to measure at scale, and is independent of ‘usual-doctor’ practice arrangements.

**Table:** How this fits in

Longitudinal continuity of care is associated with lower mortality, fewer hospital admissions, better care for chronic disease, and greater patient satisfaction. In spite of these benefits few practices measure continuity and measurement is not supported by health policy. Using the Usual Provider of Care (UPC) index this study found a strong correlation between patient measures of continuity and practice UPC scores. It illustrates GP continuity across a whole health economy and demonstrates that patient age and practice size are the strongest predictors. Improving continuity will require incentivisation, and regular measurement to support change.

The aims of the study were to:

- examine the association between the assessment of continuity by patients, in the annual GPPS, with practice consultation data using the UPC index; and
- measure longitudinal continuity of care across all practices in three contiguous boroughs in East London, and examine the variation by age, sex, ethnicity, social deprivation, and practice size.

## METHOD

### Design and setting

This was a retrospective, observational, cross-sectional study using anonymised data from the primary care records of 1.06 million adults registered with 126 practices in the three adjacent East London boroughs of Tower Hamlets, City and Hackney, and Newham. This mobile, inner-city study population includes 48% of people from ethnic minorities and is in the top decile of social deprivation in England.^21^^,^^22^

The measurement of longitudinal continuity requires a reasonably long timeframe, and a ‘run in’ period when the patient may be getting to know the practice. In common with other researchers this study used a study period of 2 years to assess continuity, and required 1 year of registration with the practice before the study period.^6^

All GP-registered patients were included if they had three or more GP consultations during the study period (1 January 2017 to 31 December 2018) and had been registered for at least 1 year before the start of the study.

### Data sources

Data were extracted on secure N3 terminals from EMIS Web, which is used by all practices in the study area. All data were anonymous and managed according to UK NHS information governance requirements.

Sociodemographic variables included age, sex, and self-reported ethnicity captured at the time of registration with the practice or during routine consultations. Ethnic categories were based on the 18 categories of the UK 2011 census and were combined into four groups reflecting the study population: White (British, Irish, other White), Black (Black African, Black Caribbean, Black British), South Asian (Bangladeshi, Pakistani, Indian, Sri Lankan, British Asian, other Asian or mixed Asian), and other (Chinese, Arab, any other ethnic group). Individuals of mixed ethnicity were grouped with their parent ethnic minority for the purposes of this study.^23^^,^^24^ The English Indices of Multiple Deprivation (IMD) 2015 score was used as a measure of social deprivation.^21^ The IMD score for each patient was mapped to the patient lower super output area of residence to derive internal and national quintiles for the study population. Consultation data included all face-to-face surgery consultations and home visits by GPs, but excluded telephone contacts. Consultations with other members of the clinical team were not included in this study. The unique numeric indicator for each doctor was used to calculate the UPC score.

### Patient experience scores

In common with the approach of previous studies, the current study included the responses from Question 10 on continuity from the annual Ipsos MORI GPPS in 2019. Question 10 asks ‘How often do you see or speak to your preferred GP when you would like to?’ The proportion of patients answering positively (‘Always, or almost always’/‘A lot of the time’) to this question, aggregated by practice, was included in the regression models. This version of the survey was chosen as the questions were answered in the months following the 2-year measurement period.

### Continuity measures

Longitudinal continuity was measured using the UPC index, defined as the proportion of contacts with the most regularly seen doctor during the 2-year study period.^16^

### Statistical analysis

All data analysis was undertaken in Stata version 16.1. Following the initial descriptive and correlation analysis, multilevel mixed-effect models nesting patients within practices were fitted. The predictors of higher scores on the UPC were examined adjusting for demographic factors and practice list size.

To ensure that the findings were not sensitive to the chosen metric a sensitivity analysis was undertaken using the Bice–Boxerman continuity of care index.

### Patient and public involvement

Patients and members of the public were not involved in the design or reporting of this study.

## RESULTS

The study population included 347 971 registered patients who contributed to the study for a full 2 years, and were registered with their GP practice for at least 1 year before the start of the study in January 2017. A flow chart detailing the case identification method is included as Supplementary Figure S1.

The mean UPC score for all 126 study practices was 0.52 (range 0.32 to 0.93). The mean UPC continuity score for practices based in the three study localities is shown in Table 1. There were marked differences in the ethnic profile and the mean list size across the three boroughs. When the three boroughs are combined >90% of the study population fall within the fourth and fifth national quintile of social deprivation

**Table 1.** Characteristics of the study population by East London borough

**Characteristic**	**All practices**	**City & Hackney**	**Newham**	**Tower Hamlets**
**Practices,** ***n***	126	42	49	35

**Number of individuals included,** ***n***	347 971	112 694	131 745	103 532

**Age, years, %**				
3–17	60 547 (17.4)	18 031 (16.0)	23 714 (18.0)	18 843 (18.2)
18–39	106 479 (30.6)	32 681 (29.0)	38 865 (29.5)	34 787 (33.6)
40–64	130 837 (37.6)	43 951 (39.0)	50 063 (38.0)	36 236 (35.0)
≥65	50 108 (14.4)	18 031 (16.0)	19 103 (14.5)	13 666 (13.2)

**Sex, %**				
Male	149 628 (43.0)	47 331 (42.0)	57 968 (44.0)	45 036 (43.5)

**Ethnicity, %**				
White	116 918 (33.6)	50 938 (45.2)	32 936 (25.0)	33 130 (32.0)
South Asian	119 354 (34.3)	10 819 (9.6)	60 339 (45.8)	48 453 (46.8)
Black	64 027 (18.4)	29 976 (26.6)	25 559 (19.4)	8593 (8.3)
Other	20 878 (6.0)	11 044 (9.8)	5270 (4.0)	3934 (3.8)
Not stated/missing	26 794 (7.7)	9917 (8.8)	7641 (5.8)	9422 (9.1)

**National IMD 2015 quintiles, %**				
1 Least deprived	2089 (0.6)	1127 (1.0)	132 (0.1)	932 (0.9)
2	4872 (1.4)	225 (0.2)	790 (0.6)	4348 (4.2)
3	19 137 (5.5)	6536 (5.8)	5665 (4.3)	5694 (5.5)
4	136 753 (39.3)	39 443 (35.0)	73 777 (56.0)	24 951 (24.0)
5 Most deprived	185 120 (53.2)	65 363 (58.0)	51 381 (39.0)	67 607 (65.3)

**List size, mean**	8842	7504	8345	9487

**UPC score, mean (SD)**	0.52 (0.11)	0.51 (0.09)	0.54 (0.13)	0.50 (0.11)

*IMD = Index of Multiple Deprivation. SD = standard deviation. UPC = Usual Provider of Care.*

**Figure 1.**
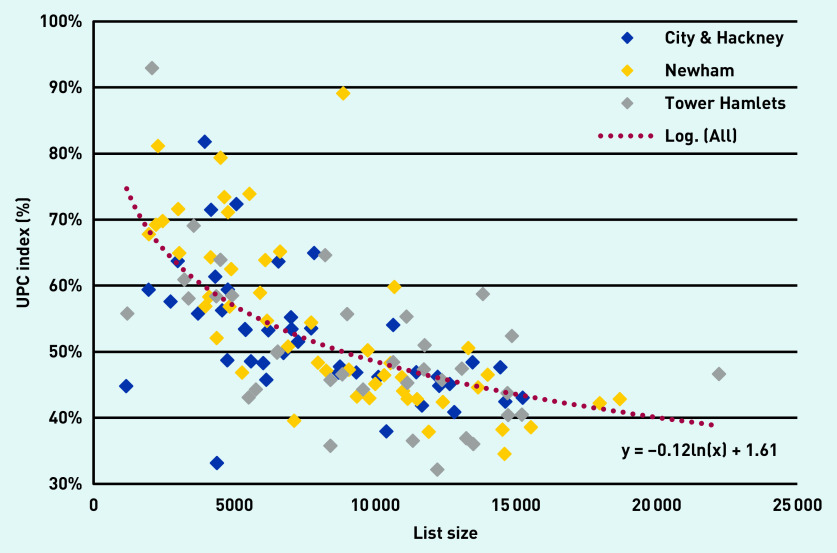
*Continuity scores (UPC) plotted against list size, for 126 GP practices in three neighbouring boroughs in East London between January 2017 and December 2018. (Each diamond represents a practice in one of the three London boroughs. The dotted line is the logarithmic trend line for all 126 practices. UPC = Usual Provider of Care.)*

The difference in continuity scores between practices, and the relationship between the UPC score and practice size, can be seen in Figure 1. This shows a similar relationship between UPC score and list size for each of the three boroughs in the study.

Sensitivity analysis showed a close correlation between the UPC and Bice–Boxerman in measurement of mean practice UPC: Pearson’s *r*, correlation coefficient 0.99.

Longitudinal continuity was examined by age group, sex, and internal quintile of social deprivation as measured by IMD score. The univariate analysis shows stepwise gains in continuity with increasing age, and greater continuity for males compared with females but no differences between the four major ethnic groups in the study area. Continuity was lowest for populations in the two most deprived quintiles (Table 2).

**Table 2.** UPC scores by age group, sex, ethnicity, and social deprivation (*n* = 347 728 patients)^a^ in the univariate analysis

**Characteristic**	**UPC, mean (SD)**
**Entire study population**	0.52 (0.11)

**Age, years**	
3–17	0.45 (0.21)
18–39	0.48 (0.22)
40–64	0.51 (0.23)
≥65	0.54 (0.23)

**Sex**	
Male	0.51 (0.23)
Female	0.48 (0.22)

**Ethnicity**	
White	0.50 (0.22)
Black	0.50 (0.22)
South Asian	0.50 (0.22)
Other	0.49 (0.22)

**Internal IMD 2015 quintile**	
1 (least deprived)	0.50 (0.23)
2	0.50 (0.22)
3	0.50 (0.22)
4	0.49 (0.22)
5 (most deprived)	0.49 (0.22)

*IMD = Index of Multiple Deprivation.*

*SD = standard deviation. UPC = Usual Provider of Care.*

a *Missing data in 243 cases restricted the number for analysis to 347 728.*

The relationship between practice UPC scores for the 2-year study period and the positive answers (‘Always, or almost always’/‘A lot of the time’) to the GPPS Question 10 ‘How often do you see or speak to your preferred GP when you would like to?’ were examined next. The 2019 GPPS results for each practice (which were recorded in the months following the study period) were linked to the UPC scores for each practice. The UPC scores and the GPPS results were found to be highly correlated: Pearson’s *r* correlation coefficient, 0.62. This indicates that patient views on continuity in a practice are closely aligned with the objective UPC score used in this study.

To investigate the predictors of continuity further a multilevel model was developed, nesting individuals within practices, and practices within boroughs (Table 3). This adjusted analysis confirms the stepwise relationship between older age groups and increased levels of continuity. In the univariate analysis male sex was associated with greater continuity, but this is reversed in the adjusted analysis where female sex has higher rates of continuity. People of White ethnicity, and groups from less deprived quintiles of the population, showed small gains in continuity.

**Table 3.** Multilevel regression analysis to identify predictors of UPC scores (*n* = 347 728 patients contributing to this model)^a^

**Variable**	**Demographic and practice factors**		

	β **coefficient**	**95% CI**	***P*-value**
**Age, years**			
3–17	Reference	—	—
18–39	0.030	0.028 to 0.032	<0.001
40–64	0.055	0.053 to 0.057	<0.001
≥65	0.082	0.080 to 0.084	<0.001

**Sex**			
Male	Reference	—	—
Female	0.026	0.025 to 0.027	<0.001

**Ethnicity^b^**			
White	Reference	—	—
Black	−0.010	−0.012 to −0.008	<0.001
South Asian	−0.016	−0.018 to −0.014	<0.001
Other	−0.004	−0.007 to −0.001	0.01

**Internal IMD 2015 quintile**			
1 (least deprived)	Reference	—	—
2	−0.005	−0.007 to −0.002	<0.001
3	−0.006	−0.008 to −0.004	<0.001
4	−0.006	−0.008 to −0.004	<0.001
5 (most deprived)	−0.009	−0.011 to −0.007	<0.001

**Practice list size**			
<5000	Reference	—	—
≥5000 to <10 000	−0.101	−0.141 to −0.062	<0.001
≥10 000	−0.181	−0.219 to −0.142	<0.001

**Locality**			
City & Hackney	Reference	—	—
Newham	0.031	−0.005 to 0.068	0.09
Tower Hamlets	0.008	−0.032 to 0.048	0.68

a *Multilevel model comprises patients nested within practices. Intraclass correlation (ICC) showing the proportion of variation in UPC scores at practice level = 0.259.*

b *Ethnicity not stated/missing is not reported. CI = confidence interval. IMD = Index of Multiple Deprivation. UPC = Usual Provider of Care.*

Practice list size is an important determinant of continuity. Small practices have the highest levels of continuity (Figure 1), but medium-sized practices (with list size from ≥5000 to <10 000 patients) also show significantly better continuity scores compared with practices with ≥10 000 patients. Once population factors, social deprivation, and practice list size have been taken into account the crude differences in UPC by borough (Table 1) are no longer significant.

## DISCUSSION

### Summary

Using practice level demographic data over a 2-year period it was possible to provide an assessment of longitudinal continuity of care for all general practices across an entire health economy. In the young, mobile, and multi-ethnic population of East London the average practice UPC score was 0.52 (SD 0.11).

The most important demographic predictor of greater continuity is the practice proportion of older patients, and the major organisational predictor is practice size, with larger practices having lower scores.

There is a strong positive correlation between the views of patients on their ability to see or speak to their preferred GP, as measured by the annual GPPS, and the UPC score for each practice during the previous 2 years.

### Strengths and limitations

This study examined continuity across a whole health economy, including all general practices in three contiguous inner-city East London boroughs. This provides a realistic assessment of inner-urban continuity in a multi-ethnic population with high levels of social deprivation, in comparison with studies that use selected practices or defined subpopulations, such as older people.

The UPC was chosen as the measure of longitudinal continuity as it has high face validity, is neutral to ‘usual-doctor’ systems within practices, and is most frequently used in comparable studies; however, a sensitivity analysis was also included using the Bice–Boxerman index, which is more sensitive to the distribution of contacts across multiple doctors, and this found the indices to be highly correlated.

Inner East London has a young and highly mobile population, and in the current study only 33% (347 791/1 063 717) of the local population was registered for long enough, and had enough consultations, to fit the study criteria. However, it is also the case that the benefits of a continuity metric will not apply to all patients, but mainly to patients who consult more frequently. This is likely to include older people, and those with multimorbidity or mental health problems. Geographic differences in local demography, including age, mobility, and social deprivation, will all affect the length of registration with a GP practice. These are external factors that limit the ability of practice teams to deliver continuity of care. When comparing studies, it is important to take such local, contextual factors into account, as well as aspects such as the continuity measurement tool, and the time period over which continuity is assessed.

The COVID-19 pandemic has accelerated changes to the way that patients and doctors interact. Remote consultations have brought convenience and speed of access for some, but may provide less personal, more transactional care. Although this is satisfactory for many problems, those with complexity and multimorbidity benefit from relationship-based care. This concept brings together patient-centred care, characterised by shared decision making and respect for patient preferences, with the notion of a therapeutic relationship. Both of these aspects of care are underpinned by longitudinal continuity.^9^^,^^25^

This study did not include telephone, video, or email-based consultation data, and did not address continuity with other clinicians such as practice nurses. These components of care will require future study.

### Comparison with existing literature

The majority of studies on continuity of care in England use data from the sample of patients who respond to the annual GPPS to assess levels of continuity. They have also used this data to demonstrate the fall in continuity over the past decade.^10^^,^^13^^,^^26^

Some previous studies have used the UPC applied to a random sample of consultations, for example, Salisbury *et al* in 2009 who showed little effect of advanced access (a systems approach that aims to see patients on the day of their choice) on continuity.^16^ Others have used a subset of the consulting population. Barker *et al* in 2017 used the UPC across multiple practices to examine the association between continuity and hospital admissions.^6^ Using a 2-year period for assessment the average UPC was 0.61; however, all the patients studied were aged >60 years — a group in which continuity is known to be higher than average. However, this figure compares favourably with the findings in the current study of a mean UPC of 0.54 for patients aged ≥65 years (Table 2) in the univariate analysis. Similarly, Sidaway-Lee *et al* in a study based in a single practice^20^ used the UPC alongside a bespoke measurement tool, and found a mean UPC of 0.61 in 2019; this compares with the current study, which found a mean practice UPC of 0.52.

This study is the first to measure longitudinal continuity across the entire population of a health district, and to compare a computerised, consultation-based measure with the practice samples surveyed by the GPPS. The correlation between these two measures provides useful validation and support for regular use of the UPC.

Up to now the diversity of methods of measuring longitudinal continuity, and the lack of published results allowing comparison across different populations, may have discouraged GPs from attempting to measure continuity. To be useful for health policy this aspect of care requires a reliable, objective tool for enabling comparison between practices, and the ability to measure changes in continuity in response to practice interventions.

### Implications for research and practice

There is increasing evidence that longitudinal continuity, used as a marker of relationship-based clinical care, provides better clinical outcomes, particularly for those who are older and those with complex problems where patient preferences and clinical judgement may trump guideline-based care.^27^

This goes against the grain of recent developments — in particular the increasing specialisation and fragmentation of primary care services, larger practices, changing professional work patterns, and the emphasis on rapid access. These can all work against valuing continuity. However, studies based on the GPPS indicate that good doctor–patient communication, rather than rapid access, is the stronger driver of patient satisfaction and that two-thirds of patients value relational continuity.^26^^,^^28^

To reverse these trends will require professional leaders who recognise that relationship continuity can no longer be taken for granted, and that GPs must play a more active role in making it possible. The authors of the current study suggest that the UPC could be considered as a new quality indicator for practices, with regular assessment on a rolling basis.

Providing resources and incentives to improve care in this way will require engagement from the emerging primary care networks and integrated care systems. Local initiatives to improve continuity, such as the development of micro-teams within larger practices^27^^,^^29^ or changes to booking systems, need to be underpinned by reliable monitoring data.

## Data

All data relevant to the study are included in the article.
